# The use of prealbumin as a predictor of malnutrition in cirrhotic patients and the effect of nutritional support in patients with low prealbumin levels

**DOI:** 10.3906/sag-1910-27

**Published:** 2020-04-09

**Authors:** Zuhal DAĞ, Hüseyin KÖSEOĞLU, Murat KEKİLLİ

**Affiliations:** 1 Department of Internal Medicine, Erol Olçok Education and Research Hospital, Çorum Turkey; 2 Department of Gastroenterology, Faculty of Medicine, Hitit University, Çorum Turkey; 3 Department of Gastroenterology, Ankara Education and Research Hospital, Ankara Turkey

**Keywords:** Malnutrition, cirrhosis, prealbumin, nutritional support, MELD score

## Abstract

**Background/aim:**

Malnutrition is an important and commonly seen prognostic factor in patients with cirrhosis. The diagnosis of malnutrition in cirrhosis patients may be challenging, and an easily measured and widely usable marker is lacking. Prealbumin, however, is an easily measured marker. In the current study we measured prealbumin levels in cirrhotic patients with no clinically apparent malnutrition and used it as a malnutrition marker. Another aim of this study was to evaluate the effect of nutritional support on patient with low prealbumin levels.

**Materials and methods:**

Fifty-two patients with Child A and Child B cirrhosis were selected for the study. Prealbumin levels were studied, and Child and MELD scores were calculated. Patients with prealbumin levels ˂180 mg/L were considered to have malnutrition, and two different types of nutritional products were given to these patients. The patients given nutritional support were investigated a month later, and parameters were compared.

**Results:**

According to the prealbumin threshold of 180 mg/L, malnutrition frequencies were 59.3% for Child A and 95% for Child B cirrhosis. After the provision of nutritional support statistically significant improvements in albumin and INR levels were detected. In addition, the MELD score decreased; however, it was not statistically significant (P: 0.088). A statistically significant decrease in the MELD score was only obtained in patients with Child B cirrhosis (P: 0.033). When the oral replacement therapies were investigated separately, a statistically significant decrease in MELD scores was detected with product 1 (P: 0.043).

**Conclusion:**

Prealbumin can be used as an easily measured parameter for earlier detection of malnutrition in patients with cirrhosis and without clinically apparent malnutrition. Oral nutritional support, especially with products containing relatively high carbohydrate levels and low protein, may have a favorable effect on MELD scores.

## 1. Introduction

Malnutrition is commonly seen in patients with cirrhosis. Most trials show that 60%–100% of patients with advanced cirrhosis have malnutrition [1–4]. Malnutrition may even be seen in patients with Child A cirrhosis, at a frequency of 25% to 46% [1,4]. Malnutrition is a poor prognostic factor in cirrhosis and is associated with lower survival and higher complication rates [5–9]. The correction of malnutrition may improve mortality, increase quality of life, decrease complications, and prepare patients for liver transplantation [6]. Malnutrition in patients with cirrhosis results from a variety of factors, including inadequate intake, poor-quality diet, malabsorption, maldigestion, altered macronutrient metabolism, and a hypermetabolic state [10].

Prealbumin is an easily measured marker for predicting protein malnutrition [11,12]. It can be synthesized by the liver up until advanced cirrhosis, and fluid retention has no effect on prealbumin [12]. After adequate nutrition is provided, prealbumin levels increase rapidly and may come to within normal range in 8 days [11]. To date, the use of prealbumin in determining malnutrition in patients with cirrhosis was not sufficiently investigated. 

Nutritional support with enteral or parenteral nutrients may be beneficial in patients with cirrhosis. The European Association for the Study of the Liver suggests nutritional counseling in cirrhotic patients with malnutrition [13]. The optimal daily energy intake should be higher than 35 kcal/kg with 1.2–1.5 g/kg of protein intake [11,14].

The aim of this study was to evaluate prealbumin as an early marker in predicting malnutrition in Child A and Child B cirrhosis without clinically evident malnutrition. Another aim was to evaluate the effect of nutritional support with oral supplements on clinical and laboratory findings in these patients. 

## 2. Materials and methods

This study was performed prospectively at Ankara Education and Research Hospital between November 2014 and May 2015. Patients who had compensated cirrhosis with different etiologies and were managed as outpatients were included in the study. The diagnosis of cirrhosis was based on a combination of clinical, laboratory, and radiologic findings, and liver biopsy was performed if the diagnosis was indefinite. Patients who were under 18 years old; who had sepsis, major psychiatric diseases, cancer diagnosis, kidney failure, alcohol abuse, decompensated cirrhosis, hepatic encephalopathy, Child C cirrhosis, or fulminant hepatic failure were excluded from the study. Patients whose body mass index (BMI) was lower than 18.5 kg/m2, and patients who were taking warfarin were not included in the study. At the beginning of the study, demographic features of the patients were recorded, and laboratory examinations were performed. Total white blood cell (WBC), hemoglobin (Hb), hematocrit (HTc), and platelet (PLT) levels were determined in samples obtained from peripheral blood; serum aspartate aminotransferase (AST), alanine aminotransferase (ALT), alkaline phosphatase (ALP), gama-glutamil transferase (GGT), albumin, total protein, sodium, potassium, calcium, blood urea nitrogen (BUN), creatinine, C-reactive protein (CRP), international normalized ratio (INR), 25 hydroxyvitamin D, vitamin B12, ferritin, thyroid-stimulating hormone (TSH), prealbumin, and alpha-fetoprotein (AFP) levels were examined with fully automated devices. MELD and Child–Pugh scores were calculated. Body mass index was calculated by dividing weight in kilograms by the square of the height in meters.

None of the patients involved in this study had clinically evident malnutrition. Patients whose prealbumin levels were ˂180 mg/L were considered to have malnutrition; these patients received oral supplementation with standard products. The patients were randomly divided into two groups, and two different oral supplements were used. Product 1 contained 1.5% kcal/mL, 16.7% protein, 53.8% carbohydrate, and 29.5% fat. Product 2 contained 1.5% kcal/mL, 22.1% protein, 47% carbohydrate, and 28.8% fat with calcium β-hydroxy-β-methylbutyrate, fructooligosaccharides, and vitamin D. Both supplements were taken at 220 mL, three times a day. After 1 month of oral supplement therapy the patients were called for control, and the same laboratory parameters were performed and body weight was assessed. 

The local medical ethics committee approved the study design and methods, and all patients provided written informed consent prior to participating in the study (medical ethics committee approval date: 05.11.2014; number: 4700). 

All statistical analyses were performed with SPSS 16.0 (IBM Co., Chicago, USA) software. The Kolmogorov–Smirnov test was used to determine the normality of the variables. Independent sample t-test, The Kruskal–Wallis test, and chi-square test of independence were used to determine the differences in variables. A paired sample t-test was used to compare variables before and after oral supplement therapy. Pearson’s correlation analysis was used to identify the correlation between parameters. P values of less than 0.05 were considered significant.

## 3. Results

Patients admitted to our gastroenterology and internal medicine clinic between November 2014 and May 2015 were included in this study. After exclusions, 32 patients with Child A cirrhosis and 20 patients with Child B cirrhosis were included for evaluation. The sociodemographic features and laboratory parameters at the beginning of the study are summarized in Table 1. 

**Table 1:** Sociodemographic features of patients with Child A cirrhosis and Child B cirrhosis.

	Child A	Child B	P
Sex (male/female)	24/8	11/9	0.224
Age, mean (years)	59.65 ± 2.43	62 ± 3.65	0.57
Height, mean (m)	166.30 ± 1.75	161.58 ± 3.36	0.54
Weight, mean (kg)	82 ± 3	75.41 ± 9	0.072
BMI, mean (kg/m2)	28.80 ± 5.45	27.19 ± 10.56	0.052
Disease duration (months)	29.26 ± 10.37	53.25 ± 18.33	0.269
Hb (g/dL)	13.46 ± 1.87	11.43 ± 3.07	0.004
PLT	140968.8 ± 73120	119650 ± 68332	0.293
INR	1.10 ± 0.13	1.34 ± 0.23	0.001
Albumin (g/dL)	4.09 ± 0.36	2.98 ± 0.44	0.001
Total bilirubin (mg/dL)	0.98 ± 0.57	1.45 ± 0.65	0.009
AST (U/L)	39.40 ± 18.60	74.00 ± 48.34	0.001
ALT (U/L)	114.62 ± 114.62	151.55 ± 74.79	0.027
25 OH vit D (µg/L)	19.22 ± 12.96	9.93 ± 6.26	0.001
Vit B12 (pg/mL)	343.71 ± 275.32	603.45 ± 342.37	0.004
Prealbumine (mg/L)	170.90 ± 49.06	86.31 ± 41.10	0.001

Body mass index, Hb: hemoglobin, PLT: platelets, AST: aspartate aminotransferase, ALT: alanine aminotransferase, 25 OH vit D: 25-hydroxyvitamin D.

The etiology of cirrhosis was hepatitis B in 22 patients (42.3%), hepatitis C in 7 patients (13.5%), alcohol in 3 patients (5.8%), autoimmune hepatitis in one patient (1.9%), and nonalcoholic steatohepatitis in one patient (1.9%). Cryptogenic cirrhosis was present in 18 patients (34.6%). 

Among the 52 patients, 38 patients had low prealbumin levels (73%). The proportions of malnourished patients (determined as low prealbumin levels) were 59.3% and 95% in patients with Child A and Child B cirrhosis, respectively. Patients with low prealbumin levels received oral nutrition therapy with oral supplements. Thirty-eight patients received oral supplements; 24 patients continued to follow-up and receive their oral supplements. Among these patients, 13 had Child A cirrhosis, and 11 had Child B cirrhosis. After 1 month of oral supplement therapy the patients were reevaluated. The laboratory parameters, Child and MELD scores, and weight values before and after therapy are given in Table 2. Statistically significant improvement was detected in INR and albumin values. The same variables were compared for patients with Child A and Child B cirrhosis separately (Table 3). No statistically significant change was detected in patients with Child A cirrhosis, but statistically significant increases in albumin and statistically significant decreases in INR and MELD scores were detected in patients with Child B cirrhosis. 

**Table 2:** Laboratory parameters, weight values, and Child and MELD scores in patients before and after nutritional support with both oral supplements.

	Before therapy	After therapy	P
Weight, mean (kg)	79.83 ± 24.11	80.47 ± 24.43	0.334
BMI, mean(kg/m2)	29.06 ± 10.00	29.28 ± 10.06	0.373
Hb (g/dL)	13.05 ± 2.49	13.20 ± 2.36	0.666
PLT	108540 ± 62205	97750 ± 55401	0.088
INR	1.28 ± 0.24	1.18 ± 0.20	0.012
Albumin (g/dL)	3.58 ± 0.55	3.78 ± 0.60	0.025
T bilirubin (mg/dL)	1.37 ± 0.63	1.40 ± 0.78	0.741
AST (U/L)	59.04 ± 33.01	51.87 ± 27.09	0.307
ALT (U/L)	51 ± 33.89	44.37 ± 28.37	0.257
Prealbumin (mg/L)	111.62 ± 42.26	122.43 ± 46.20	0.118
Child Score	6.33 ± 1.40	6.17 ± 1.24	0.588
MELD score	10.17 ± 3.25	9.50 ± 2.87	0.088

BMI: Body mass index, Hb: hemoglobin, PLT: platelets, AST: aspartate aminotransferase, ALT: alanine aminotransferase.

**Table 3:** Laboratory parameters, weight values, and Child and MELD scores in patients before and after nutritional support with both oral supplements for patients with Child A cirrhosis and Child B cirrhosis, separately.

	Child A			Child B		
	Before therapy	After therapy	P	Before therapy	After therapy	P
Weight	83.85 ± 14.21	83.54 ± 14.54	0.746	75.09 ± 32.40	76.84 ± 33.05	0.052
BMI	30.19 ± 14.54	30.08 ± 5.06	0.753	27.71 ± 13.99	28.33 ± 14.15	0.056
Hb (g/dL)	14.22 ± 1.02	13.99 ± 1.88	0.659	11.68 ± 3.02	12.25 ± 2.59	0.167
PLT	126615 ± 68870	112307 ± 66637	0.190	87181 ± 47767	80545 ± 33610	0.258
INR	1.12 ± 0.12	1.08 ± 0.12	0.081	1.46 ± 0.20	1.30 ± 0.21	0.037
Albumin (g/dL)	4.00 ± 0.32	4.07 ± 0.52	0.501	3.08 ± 0.25	3.44 ± 0.51	0.020
T bilirubin (mg/dL)	1.02 ± 0.49	1.07 ± 0.66	0.564	1.79 ± 0.53	1.79 ± 0.76	0.981
AST (U/L)	46.77 ± 19.66	43.54 ± 16.12	0.444	73.54 ± 40.19	61.73 ± 34.34	0.432
ALT (U/L)	47.46 ± 26.38	39.77 ± 16.93	0.266	55.18 ± 42.08	49.82 ± 38.05	0.606
Prealbumin (mg/L)	140.45 ± 26.29	145.35 ± 47.95	0.628	77.54 ± 30.32	95.35 ± 25.69	0.068
Child Score	5.23 ± 0.44	5.31 ± 0.48	0.337	7.64 ± 0.92	7.18 ± 1.08	0.138
MELD score	7.92 ± 1.98	8.00 ± 2.38	0.829	12.82 ± 2.32	11.28 ± 2.41	0.033

BMI: Body mass index, Hb: hemoglobin, PLT: platelets, AST: aspartate aminotransferase, ALT: alanine aminotransferase, 25 OH vit D: 25-hydroxyvitamin D.

Eleven of the patients received product 1, and 13 patients took product 2 as an oral supplement. The patients were evaluated separately for product 1 and product 2, and variables before and after therapy are summarized in Table 4.

**Table 4:** Laboratory parameters, weight values, and Child and MELD scores in patients before and after nutritional support with each oral supplement.

	Product 1			Product 2		
	Before therapy	After therapy	P	Before therapy	After therapy	P
Weight	76.82 ± 16.63	76.94 ± 16.06	0.892	82.38 ± 29.47	83.46 ± 30.13	0.286
BMI	28.14 ± 6.74	28.15 ± 5.27	0.968	29.84 ± 12.77	30.23 ± 12.99	0.280
Hb (g/dL)	13.18 ± 2.17	12.47 ± 2.45	0.163	12.95 ± 2.81	13.81 ± 2.18	0.029
PLT	115545 ± 66643	107363 ± 72212	0.237	102615 ± 60271	89615 ± 37160	0.216
INR	1.28 ± 0.28	1.14 ± 0.16	0.012	1.28 ± 0.21	1.22 ± 0.22	0.259
Albumin (g/dL)	3.60 ± 0.52	3.61 ± 0.59	0.931	3.56 ± 0.59	3.93 ± 0.59	0.002
T bilirubin (mg/dL)	1.43 ± 0.67	1.54 ± 1.03	0.420	1.33 ± 0.63	1.28 ± 0.50	0.580
AST (U/L)	49.81 ± 17.88	53.36 ± 34.42	0.618	66.85 ± 40.97	50.51 ± 20.38	0.161
ALT (U/L)	40.82 ± 19.16	42.36 ± 28.59	0.808	59.62 ± 41.47	46.08 ± 29.24	0.155
Prealbumin (mg/L)	112.67 ± 46.68	111.15 ± 42.34	0.860	110.72 ± 40.06	131.97 ± 48.81	0.043
Child Score	6.18 ± 1.40	6.27 ± 1.56	0.588	6.46 ± 1.45	6.08 ± 0.95	0.096
MELD score	10.27 ± 3.55	9.36 ± 3.47	0.043	10.08 ± 3.12	9.62 ± 2.39	0.468

BMI: Body mass index, Hb: hemoglobin, PLT: platelets, AST: aspartate aminotransferase, ALT: alanine aminotransferase, 25 OH vit D: 25-hydroxyvitamin D.

After therapy, prealbumin levels increased to normal levels in only 4 patients. All of these patients had Child A cirrhosis; none of the patients with Child B cirrhosis had normal prealbumin levels after therapy. Because of the low number with normal prealbumin levels after therapy, statistical analysis was not performed between patients with and without normal prealbumin levels after therapy. Among the patients, 15 had an increase in prealbumin levels, whereas the prealbumin levels of 9 patients decreased. When patients with and without prealbumin increases were compared, there was no difference in laboratory parameters between the two groups, with the exception of prealbumin, which was lower in patients who had an increase in prealbumin levels (99.42 ± 45.77 vs. 131.94 ± 26.91, P: 0.039). 

## 4. Discussion

Malnutrition is a commonly seen and poor prognostic factor in patients with cirrhosis [1,5]. It is associated with higher complication rates and lower survival in patients with cirrhosis [5–9]. It is important to recognize malnutrition in such patients. However, detecting malnutrition in patients with cirrhosis is not always easy due to the presence of ascites and fluid retention. Body mass index alone may not be sufficient for detecting malnutrition in patients with cirrhosis. Cross-sectional imaging, like CT, allows the direct quantification of muscle mass and may be used for the assessment of malnutrition [15,16]. CT imaging may help to diagnose malnutrition in cirrhosis, but its routine use is limited in clinical practice because of cost, time, and radiation exposure [13]. Anthropometry, DEXA, and BIA are alternative diagnostic methods for malnutrition [13]. Anthropometric evaluation, DEXA, and BIA also have limitations for the detection of malnutrition. For these reasons an easily accessible diagnostic marker for the prediction of malnutrition is needed. 

Prealbumin is an easily measured marker for protein malnutrition. The use of prealbumin for detecting malnutrition in patients with cirrhosis has not been widely investigated. Our study evaluated serum prealbumin levels for the diagnosis of malnutrition in patients with no clinically apparent malnutrition. The aim of this approach is to detect malnutrition in the early phases, especially before clinical findings appear. The frequency of malnutrition, defined as low prealbumin levels, was 73% in our study population. Because our study population contained only compensated cirrhotic patients with no clinically apparent malnutrition, these results show that malnutrition is much more common than predicted. Although the use of prealbumin for detecting malnutrition in cirrhotic patients is not well validated, the fact that prealbumin can be synthesized by the liver up until advanced cirrhosis [12] indicates that it could be used as a malnutrition marker in compensated cirrhosis. The easy use and low expense of prealbumin could make it a useful malnutrition marker in patients with early cirrhosis and without clinically apparent malnutrition.

Oral intake of food is the preferred way to meet nutritional requirements; however, this intake may be insufficient in cirrhotic patients [14]. Nutritional support with oral supplements may provide alternative nutrition in patients with malnutrition and cirrhosis. It has been shown that oral supplements are effective in hospitalized patients [15]. The effect of oral supplements on the natural history of cirrhosis is not well established, and a limited number of studies have been published to date. Hirsch et al. showed that nutritional supplementation reduces hospitalization for complications in patients with alcoholic cirrhosis, but they could not show any benefit in liver function parameters after 1 year of nutritional support [17]. It has been shown that enteral nutrition has favorable effects in severely malnourished cirrhotic patients [18] and in patients with alcoholic liver disease [19]. However, other studies showed no benefit of enteral nutrition in liver diseases [20,21]. Our study is different from these trials, because none of these studies included cirrhotic outpatients without clinically significant malnutrition. Another difference is that our study population consisted of patients with cirrhosis of different etiologies. The aforementioned studies included patients with alcoholic liver diseases [18,20,21]; in our study alcohol was the etiology for cirrhosis in only a minority of patients. 

To the best of our knowledge the effect of nutritional support with oral supplements in cirrhotic patients with no clinically apparent malnutrition has not been studied, and we evaluated two different oral supplement products in this study population. Patients, whose prealbumin levels were ˂180 mg/L received two different types of oral supplementation after randomization. The first product contained more carbohydrates, whereas the second product contained more protein and included calcium β-hydroxy-β-methylbutyrate, fructooligosaccharides, and vitamin D. The European Association for the Study of the Liver suggests nutritional counseling in cirrhotic patients with malnutrition [13]. The optimal daily energy intake should be higher than 35 kcal/kg with 1.2–1.5 g/kg of protein intake in 3 main meals and 3 snacks [11,14]. According to this suggestion the goal for calorie intake was 35 kcal per kg weight. After 1 month with oral supplementation statistically significant improvement in albumin and INR levels were obtained. After therapy the mean Child and MELD scores decreased; however, statistical significance could not be achieved. Significant decreases may be achieved with a larger study population and longer duration of oral supplement support. A statistically significant decrease in MELD scores was achieved in patients with Child B cirrhosis. This shows that oral nutritional support is effective in patients with Child B cirrhosis and no clinically apparent malnutrition. 

When the products were evaluated separately, significant increases in prealbumin and albumin levels were achieved with product 2, and significant improvement of MELD scores and INR levels were detected with product 1. These data show that higher levels of protein in the oral supplements may improve prealbumin and albumin levels, but this has no favorable effect on MELD scores. The exact mechanism of this discrepancy is unknown, but these findings suggest that the decrease in MELD scores is not due to the increase in prealbumin levels. The positive effect of product 1 on MELD scores shows that calories derived from carbohydrates should comprise at least 50% of total calorie intake. Our study also showed that adding calcium β-hydroxy-β-methylbutyrate, fructooligosaccharides, and vitamin D has no favorable effect over calorie support alone. Similarly, a recently published metaanalysis does not show any benefit of vitamin D supplementation on NAFLD treatment [22].

The current study has limitations. The low number of patients in our study population is a major limitation. In addition, we provided 1 month of nutritional support, but a longer duration of supplementation may be more effective. With a higher number of study participants and increased duration of nutritional support the differences detected in MELD and Child scores may reach statistically significant values. In the current study we used prealbumin as a predictor for malnutrition in patients with no clinically apparent malnutrition. The collection of CT, DEXA, or BIA values at the beginning of the study and a comparison of these values with prealbumin levels would be valuable; however, the routine use of these methods is not recommended, and could include ethical issues. However, these limitations do not hinder the results of the study. We showed for the first time that oral nutritional support improves MELD scores in a group of patients with cirrhosis and without clinically apparent malnutrition, especially in patients with Child B cirrhosis. We suggest that prealbumin levels may be used as an early indicator of malnutrition, and patients with low prealbumin levels may benefit from oral nutritional support. 

There is a strong association between cirrhosis and malnutrition. Prealbumin levels may be helpful for detecting malnutrition in cirrhotic patients who have no clinically apparent malnutrition. Ease of measurement and low cost are advantages of prealbumin. Oral nutritional support showed some beneficial effects in patients with low prealbumin levels. An INR decrease and albumin increase was obtained in the study population as a whole, and a decrease in MELD scores was achieved in patients with Child B cirrhosis. Different results were achieved with two different products, and a statistically significant decrease in MELD score was obtained with product 1. Product 1 contained 1.5% kcal/mL: 16.7% protein, 53.8% carbohydrate, and 29.5% fat. More significant results may be found with higher numbers of study participants and nutritional support of longer duration. 

## References

[ref0] (2005). Nutritional state and energy balance in cirrhotic patients with or without hypermetabolism. Multicentre prospective study by the Nutritional Problems in Gastroenterology section of the Italian Society of Gastroenterology (SIGE). Digestive and Liver Disease.

[ref1] (2003). Evaluation of nutritional practice in hospitalized cirrhotic patients: results of a prospective study. Nutrition.

[ref2] (1984). Protein-calorie malnutrition associated with alcoholic hepatitis. Veterans Administration Cooperative study group on alcoholic hepatitis. The American Journal of Medicine.

[ref3] (2006). Evaluation of nutritional status of nonhospitalized patients with liver cirrhosis. Arquivos de Gastroenterologia.

[ref4] (2012). Muscle wasting is associated with mortality in patients with cirrhosis. Clinical Gastroenterology and Hepatology.

[ref5] (2006). Nutritional status and prognosis in cirrhotic patients. Alimentary Pharmacology & Therapeutics.

[ref6] (2011). Protein energy malnutrition predicts complications in liver cirrhosis. European Journal of Gastroenterology & Hepatology.

[ref7] (2010). Cirrhotic patients are at risk for health care-associated bacterial infections. Clinical Gastroenterology and Hepatology.

[ref8] (2013). Muscle depletion increases the risk of overt and minimal hepatic encephalopathy: results of a prospective study. Metabolic Brain Disease.

[ref9] (2015). Importance of malnutrition in patients with cirrhosis. The Turkish Journal of Gastroenterology.

[ref10] (1995). Nutritional assessment (protein nutriture). Analytical Chemistry.

[ref11] (1996). Outcomes of continuous process improvement of a nutritional care program incorporating serum prealbumin measurements. Nutrition.

[ref12] (2019). European Association for the Study of the Liver. EASL Clinical practice guidelines on nutrition in chronic liver disease. Journal of Hepatology.

[ref13] (2017). Optimizing the nutritional support of adult patients in the setting of cirrhosis. Nutrients.

[ref14] (2013). Comparison of three interventions in the treatment of malnutrition in hospitalised older adults: a clinical trial. Nutrition & Dietetics.

[ref15] (2019). A North American expert opinion statement on sarcopenia in liver transplantation. Hepatology.

[ref16] (1993). Controlled trial on nutrition supplementation in outpatients with symptomatic alcoholic cirrhosis. JPEN Journal of Parenteral and Enteral Nutrition.

[ref17] (1990). Effect of total enteral nutrition on the short-term outcome of severely malnourished cirrhotics. Gastroenterology.

[ref18] (1992). Accelerated improvement of alcoholic liver disease with enteral nutrition. Gastroenterology.

[ref19] (1989). Nutritional support in hospitalized patients with alcoholic liver disease. European Journal of Clinical Nutrition.

[ref20] (1985). Controlled trial of nutritional supplementation, with and without branched chain amino acid enrichment, in treatment of acute alcoholic hepatitis. Journal of Hepatology.

